# Students with specific learning disabilities experiences of pre-registration physiotherapy education: a qualitative study

**DOI:** 10.1186/s12909-019-1913-3

**Published:** 2019-12-31

**Authors:** M. Norris, J. Hammond, A. Williams, S. Walker

**Affiliations:** 1 Department of Clinical Sciences, College of Health and Life Sciences, Brunel University London, Kingston Lane, Uxbridge, UB8 3PH London, England; 2 Faculty of Health, Social Care and Education St George’s University of London / Kingston University, Cranmer Terrace, London, SW17 0RE England; 3 School of Health Sciences, University of Brighton, Eastbourne, BN20 7UR England

**Keywords:** Physiotherapy, Student, Disability, Attainment inequalities, Qualitative

## Abstract

**Background:**

Attainment gaps for students with disabilities have been noted in pre-registration physiotherapy courses in the UK. Previous research suggests disclosure, lack of staff knowledge and poor communication between University and placement sites may be relevant, but these are limited to small case studies with students with visual or physical disabilities. The purpose of this study was to explore disabled physiotherapy students’ experiences of their education in order to elucidate factors that may influence success.

**Methods:**

Qualitative study drawing on phenomenological traditions. Four focus groups including 15 students with disabilities were conducted. Transcripts were analysed thematically. Procedures for transparency and rigour such as member checking and peer debriefing were implemented.

**Results:**

Three major themes were derived from data. “It was quite a relief” explores the personal and social implications of diagnosis. “They’re not natural” focuses on academic assessment and the specifics of adjustments made and not made within that context. “My dyslexia doesn’t switch off” explores the inaccessibility of the learning environment and dissects the contrast between the 24-h nature of having a specific learning condition and the somewhat piecemeal nature of adjustments during their education.

**Conclusions:**

This study indicates that having a specific learning disability or anxiety creates a number of hurdles to success in physiotherapy education. Most were within the University setting and were perceived to result from staff ignorance or piecemeal approaches to inclusion. A lack of consistency alongside facilitated dialogue and acknowledgement of enhancements results in frustration, ambiguity towards disclosure and reinforcement of a deficit model. Such an approach belies the intention of the profession and the NHS and does not maximise the potential of widening participation.

## Background

There has been a specific focus on widening participation to higher education in the United Kingdom (UK) and worldwide since the beginning of the twenty-first century. Initial focus in the UK was on improving access and increasing participation for all [1, 2]. However, in the last decade evidence has emerged of attainment inequalities [3]. Hence attention has been drawn to not only making higher education accessible to all, but also ensuring that those who enter successfully complete their course of studies. This has been bolstered by the Equality Act published in 2010 [4], and specific action to monitor metrics of learning gain through the Teaching Excellence Framework [5], practices to enable equal access and appropriate support for all.

Disability is one area where attainment inequalities have been found within higher education. This is of specific relevance to physiotherapy, as 12% of pre-registration students across all UK Higher Education Institutions (HEIs) declared a disability in 2016–17 on entry to their course [6], with the majority reporting dyslexia or other specific learning disabilities. This is equivalent to national data for all courses in 2016–17 [7]. The physiotherapy profession has a long history of including people with disability within education and the workforce, in particular people with visual impairment [8]. This is supported by the National Health Service (NHS) which has stated the aim of employing people with disability [9]. However, a number of issues have been raised within the literature.

First is a recent study which demonstrates an attainment gap exists for pre-registration physiotherapy students with a disability, particularly those studying on pre-registration MSc courses [10]. While this study in physiotherapy mirrors a pattern in medicine, dentistry and higher education more generally [11], it did not explore potential reasons for attainment inequalities; other related literature in physiotherapy may give some indications. Reluctance to disclose disability before, during and after clinical training has been highlighted as an issue due to concerns with negative judgement and prejudice [8, 12, 13]. Likewise, poor levels of staff knowledge and familiarity with specific disabilities, has been linked with reduced acceptability and unsupportive behaviour [14]. This has specifically been related to placements where communication between sites (clinical and academic personnel) has not been optimised [12].

Research in higher education in other clinical professions is more prolific and highlights additional concerns such as the construction of disability within education generally and the consequential focus on ‘adjustments’ in a concessionary structure [15]. Stigma, marginalization and discrimination have been noted, both directly in universities and with clinical staff but also indirectly through the regulatory frameworks related to fitness to practice [16–19]. In contrast, studies also highlight agency of students with disabilities with potential enhancement as a consequence of living with a disability [15, 20]. To date it is not explicitly known how these relate to the broad experience of physiotherapy education, or disabilities such as dyslexia. Likewise, while there are a number of recommendations for inclusive education [21], it is not clear how these are being implemented and perceived in physiotherapy.

To complicate things further, the term disability itself is debated. We have approached this research with an awareness of current debates, but without a specific lens, remaining open to the various narratives of the participants. Given the literature on stigma and discrimination [8, 12, 13, 16, 17], we are mindful of the social model, where focus is directed towards barriers created by society [22]. However, in-keeping with critiques of this model we acknowledge that a focus on society should not detract from a need to consider the individual and their specific situation which is strongly supported in the limited physiotherapy literature [8, 23]. Furthermore, recognizing the narratives on agency and enhancements [15, 20] draws attention to the affirmative model in which the celebration of positive social identity and ownership of impairment is highlighted [24]. In contrast, Campbell’s [25] call for a more radical refocusing of disability studies that critiques the processes and practices that support and perpetuate ableism as homonormativity within society, requires a consideration of how those processes and practices may play out in physiotherapy education. This is in part reflected by Bryne [26] who reminds us to remain vigilant to the dominant discourse of ability in which ‘support’ and ‘adjustment’ are considered both as unfluctuating and as a concession which have to be earned through assessment and evidence.

Within this context, the purpose of this study was to explore disabled physiotherapy students’ experiences of their education in order to elucidate what factors might be relevant in student success. Due to the dearth of literature in physiotherapy it was impossible to predict which factors (social/environmental/individual) are more prominent in the lived experiences of physiotherapy students. Therefore, the study purposefully focused on the broad educational experience of the participants so that any factors relevant to them could be raised.

## Methods

This was a qualitative study informed by phenomenological traditions [27], in-keeping with the focus on the students’ experiences of life as a student physiotherapist with a disability.

For practical reasons two HEI’s in the South East of England were chosen to conduct the study. The total pool of students studying BSc and MSc pre-registration courses was approximately 500, therefore there were approximately 60 potential participants based on the 12% prevalence estimate of physiotherapy students with a disability previously indicated [6]. Students at the two HEI’s were invited to participate in the study via a cohort wide email. Inclusion criteria included current students, who had completed both academic modules and clinical placement and who had a confirmed disability (self-declared but also documented in University records). To maximize inclusion further sampling restrictions were not included. This can be considered a convenience sample [28] of those with the relevant experience to respond to the study aims.

Focus groups were selected given their ability to support collective responses and increased depth often prompted through interaction. A focus group at the penultimate (MSc yr 1 and BSc yr 2) and final (MSc yr 2 and BSc yr3) year level were conducted at each HEI to explore the shared phenomenon of physiotherapy education. They followed a topic guide developed in relation to previous literature and discussed and agreed by the research team (Additional file 1). It included opportunities for participants to discuss their identity, positive and more challenging experiences in university and placement, garner their thoughts on the previous study that showed an attainment gap [10] and opportunities for enhancement. Open questions were intentionally used in order to avoid leading participants in particular *apriori* directions.

All focus groups were facilitated by experienced qualitative researchers from outside the host institution, and therefore were previously unknown to the participants, who were also physiotherapy faculty members (MN with AW as co-facilitator, JH with SW as co-facilitator). One had a disability which was shared with the participants in order to enhance an atmosphere of openness. The focus groups were audio-recorded and key points were noted on flip charts during the focus groups to aid in collective member checking of the data. This was deemed particularly important as all researchers are academic faculty and these checks served to highlight potential presuppositions of priority areas.

## Analysis

**Fig. 1:**
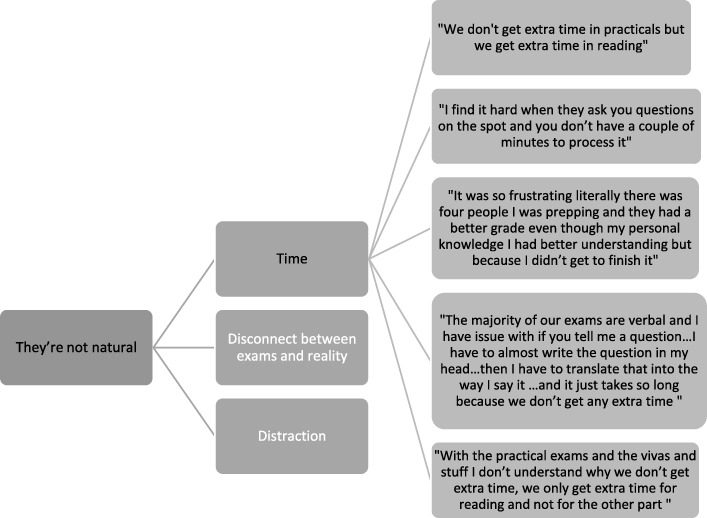
Example thematic tree

Audio-recordings were transcribed verbatim and anonymised. They were analysed thematically by one lead researcher [29]. This followed a process of familiarisation through reading of the transcripts, detailed inductive line coding and iterative development of categories and super-ordinate themes. An example of a thematic tree is given in Fig. 1. Negative case analysis was conducted to check thematic development. Three co-researcher’s read the transcripts closely and engaged in critical discussions with the lead researcher to enhance depth of analysis and transparency. One of these researchers has a specific learning disability which offered the opportunity to consider a perspective from inside the community and to challenge potential emergence of ableist discourse.

## Results

The sample included 15 students (11 F, 4 M, 12 BSc, 3 MSc) with a range of predominantly specific learning disabilities including dyslexia, anxiety, dyspraxia, Attention Deficit Disorder -ADD and Attention Deficit Hyperactivity Disorder - ADHD (Table 1). While one student with physical disability volunteered, they were unable to attend the focus group. The focus groups lasted on average 92 min (range 83–99).

**Table 1:** Participant characteristics

Participant number	FG	Gender	Course	Year of study	Self-identified disability	Point of diagnosis
F1	1	F	BSc	3	Dyslexic	1st year
F2	1	F	BSc	3	Dyslexic	During previous degree
M1	1	M	BSc	3	Dyspraxic	1st year
M2	1	M	BSc	3	ADHD	During previous degree
M3	2	M	BSc	2	Dyslexia ADD	During 1st degree
F3	2	F	MSc	1	Dyslexia	High school
F4	3	F	BSc	3	Dyslexia	1st year BSc
F5	3	F	BSc	3	Dyspraxia/nocturnal seizures	1st year BSc
F6	3	F	BSc	3	Anxiety/Sensory defensiveness	3rd year BSc
M4	3	M	MSc	2	Dyslexia	During 1st degree
F7	3	F	MSc	2	Dyslexia/Anxiety	During 1st degree
F8	4	F	BSc	2	Dyslexia	Since school
F9	4	F	BSc	2	Anxiety	After 1st degree
F10	4	F	BSc	2	Dyslexia	1st year BSc
F11	4	F	BSc	2	Dyslexia/dyspraxia	During 1st degree

Three major themes were derived from the focus group data. The first theme “it was quite a relief” explores the personal and social implications of diagnosis on a bedrock of previous hard work and social ignorance. “They’re not natural” focuses on academic assessment and the specifics of adjustments made and not made within that context. The final theme “My dyslexia doesn’t switch off” highlights aspects of education delivery which are not ideal to access learning. Students noted that while these issues would resonate with most students, they had particular significance for those with specific learning conditions. These illustrate the contrast between the 24-h nature of having a specific learning condition and the somewhat piecemeal nature of adjustments during their education. This theme ends with a clear call for institutional practices to support rather than hinder the self-management strategies that the students own. On the basis of the experiences discussed in the focus groups, students suggested specific recommendations to education and practice. These are presented in the discussion.

### “It was quite a relief”: the personal and social implications of diagnosis

As a starting point the students shared their own personal histories of struggle. Most had completed the majority of their earlier education without any formal diagnosis and therefore described how they had often felt isolated and somewhat confused by the difficulties they faced as this female participant describes:

“I was diagnosed with dyslexia when I was 19. Beforehand I’d struggled all the way through…so I was just average but working extremely hard to be average…I think if you don’t know …then you feel like you’re struggling by yourself…no one understands” F3 FG2

One student related how this lack of understanding of herself and her condition impacted on her emotionally resulting in a diagnosis of anxiety at an earlier age. Yet these are students that had succeeded in their education despite this lack of knowledge and support and the sense they were working harder than their peers in order to achieve resonated through all focus groups.

While diagnosis came late for the majority and the categorisation as having a disability itself was not unproblematic, when it came it was met with a degree of relief:

“I quite like it [diagnosis]…it was quite a relief…it justifies things…I could identify an issue” They later continue, “it’s kind of something to make me aware, I’ve got an idea of how I think and I’ve got some ideas of where I should go with it” M1 FG1

Diagnosis was usually accompanied with detailed reporting of their own specific condition. As the participant above indicates this knowledge led to understanding and a re-framing of their previous struggles. Participants talked about a sense of empowerment, awareness and insight which ultimately led to clearer ideas of personal action and acceptance which this example shows:

“once I understood it, it kind of made me feel a lot better about myself and just umm, those negative emotions and feeling they kind of dissipated a lot over time, it’s cos I understood better myself.” M2 FG1

The positive benefits of detailed reporting were noted particularly when they were accompanied by a clear and relevant introduction to strategies and how they could be implemented in real life. For one student this came through work alongside an Occupational Therapist, while another noted the usefulness of a report which specifically focused on physiotherapy related skills.

This reframing of their past history led to an increase in confidence but also appreciation of the positive traits. The students discussed their increased capacity to problem solve, be creative with ideas, look outside the box and have empathy with people who struggle, all skills which they saw as relevant additions to their future careers as therapists. This personal understanding was not present for all, influenced by the timing of their diagnosis, or the recent onset of new challenges such as placement. For those most recently diagnosed (within the last 12 months), their strategies were still developing and the work of re-understanding and reframing was still in progress.

The majority of the participants were not diagnosed until University (current or previous degree), which raised the issue of identification processes. The driver to seek testing came from a number of sources: previous work colleagues, medical personnel, personal tutors, a generic talk at induction, advice from other students and placement educators. While all were grateful for the prompt, and the awareness of role models within the profession was noted particularly favourably, the haphazard way in which it occurred was the source of concern for the participants. Students strongly expressed the need for early identification to maximise their capacity on the course which they indicated required more robust and reliable processes of identification.

“I suppose one of the really key things is just getting diagnosed like early, so you can get support in place, I suppose that's quite a major part” F10 FG4

Early identification and diagnosis, for many, lay in better awareness of the conditions. The students discussed many misconceptions they held prior to their own diagnosis, relating conditions such as dyslexia with lower intelligence. But while their own understanding developed, there were significant concerns raised about others in society, including in the academic arena. Some students reported not declaring their disability on their application for fear of judgement as a ‘problem’. This also extended to disclosure on placement through concerns of being treated differently or being supervised by staff who are “not gonna know where to start with you” F2 FG1. Mostly though, the students described positive experiences on placement with staff who were well informed or indeed had specific learning difficulties themselves. The majority of the issues raised were within the Universities themselves and specifics of this will be discussed in the following themes.

Overall this theme illustrates the complex journey students with disability have in relation to diagnosis and subsequent understanding of self. They highlight the importance of early and consistent identification which requires better awareness of conditions and signposting.

### “They’re not natural”: the false glare of assessment

This theme unpacks the assessment process and how students perceive their disability within that specific context. While the participants on the whole were appreciative of adjustments made to exams, their insights draw on potential misconceptions about their conditions resulting in apparently random decisions on adjustments to assessment.

Of particular concern were practical exams and participants consistently raised their frustration and confusion over the lack of adjustment to these assessments. A key feature of this was a lack of adjustment in time. While it was inconsistent, most described additional time for reading components of practical exams but nothing for the actual delivery of the practical skill and discussion. For them, this separation between reading and oral/aural/practical components made little sense when they had recognised problems with processing and organising information. For them this was often harder in aural/oral form then in written, which led to high levels of anxiety about the questioning processes in such exams as this example demonstrates:

“I find it hard when they ask you questions on the spot and you don’t have a couple of minutes to process it…I find if I take a couple of minutes I can produce quite an outstanding answer, but if you want answer like that, which in the exam conditions you just kind of go blurgh” F3 FG2

The anticipation of facing these exams without additional time created a vicious circle for these students. Knowledge of their own challenges with processing and delivery, led to fear of not completing the exams on time. This created anxiety and pressure to speed up, which resulted in more errors and heightened awareness of their difficulties.

A further challenge was distraction within their exam. For written assessments they were often in a room with few students or on their own. However, with practical exams they were with other students with several exams happening simultaneously. While they appreciated the complexity of organising such exams, this format resulted in a mix of distraction and pressure. The noise of others speaking and moving disturbed their focus, already a recognised challenge for many, and as a result they had to regather their thoughts, ask for questions to be repeated or misunderstood what was being asked of them. All were seen to contribute to additional stress, loss of time, but also the potential of responding to what they thought they had been asked rather than the actual question/task.

“I’ve had to ask a number of times for them to repeat the question to me because I’m fixing on something else, or like I hear a beep or something’s happening and they’ve asked me a question and cos you’re under pressure and time, I’ve suddenly forgotten, I wasn’t even listening to the question and that, I then need to ask, “Oh sorry, can you repeat that again?” F1 FG1

The participants in these focus groups frequently commented that the problems they faced were common to all students, but highlighted that they were exacerbated when you had a specific learning difficulty.

Many had raised their concerns about the lack of adjustments with academic tutors and had been told that exams were designed to mimic the clinical environment. However, this contrasted with experiences on placement where they described a greater willingness of colleagues to consider adjustments as demonstrated here:

F10: “I asked about that first year for my bad auditory memory for practical exams and they said to me, it’s not like the actual setting of being on placement…but I got extra time for my initial assessments [on placement]…When you’re here (university) they’re sort of well that’s not how it’s gonna be in real life”

F8: “but you’re learning, there’s a difference…we’re learning at uni so we should get extra time…which is why we get extra time on placement” FG4

They highlighted numerous cases where exam conditions created problems which were never replicated on placement. Time was one factor, as students described a number of placements which considered additional time to be a holistic requirement not just for writing notes. Similarly, instructions and feedback were adjusted to be given on paper rather than verbally to assist with processing and retention. Likewise, students were encouraged to create templates as memory assistance while on placement, whereas in exams they were entirely reliant on immediate recall. Mostly though it was the pressure of exams and the intense time limited focus on the student. This restriction was not perceived in clinical practice leading the participants to conclude that the assessment process placed a false glare on their abilities.

“Again it’s not natural, when you do it you’re in a placement on a ward, it’s so natural you just do it whereas in an exam in the OSCEs [Objective Structured Clinical Examination] you, as I said, it just doesn’t feel…“F3 FG2

This theme highlights a clear perceived discrepancy between how practical assessments operate within the university environment, which are not replicated within the clinical field. The complex processing and problem solving required within case based practical exams appeared not to be considered, coupled with limitations placed on strategies participants were encouraged to develop when working clinically.

### “My dyslexia doesn’t switch off”: inaccessible learning environments and piecemeal approaches to disability support

In the previous theme, an acceptance that some experiences may parallel those of non-disabled students became apparent. This next theme dissects the minutiae of the learning experience, unpacking features that create general inaccessibility, but which further impact on students with specific learning difficulties. This highlights a perceived piecemeal approach to support which contrasts with the 24 h experience of living with a disability.

Students consistently flagged the lecture arena as a complex minefield to navigate. While they acknowledged their lecturers were generally knowledgeable and engaging, the forum of the lecture, it’s structure, pace and density of material, as well as length were seen as a significant obstacle to learning. Students referred to ‘zoning out’ or metaphorically ‘leaving the room’ while remaining in it as words washed over them. The result was frustration, not only in their lack of understanding but also the awareness that they had lost time which they would have to compensate for later.

“I leave a lecture and I can hear some people being like “oh that’s really interesting” and I’m like you heard that? When was that mentioned 'cos I’ve not picked that up?...because to me it’s like someone throwing stuff at me, but in no particular order for me to actually contemplate or even put down on paper what they’ve said…it’s a bit of a set back for me ‘cos I feel like now I’ve got to go and do double the work to try and actually understand what that hour lecture was actually about” F2 FG1

A specific challenge was the necessity to write notes while listening and trying to understand. This was deemed essential as the students perceived many lecturers left slides minimally populated. This was matched with a perceived delay in posting slides prior to lectures so participants had inadequate time to familiarise themselves with the material to ease the complexity of the lecture format. Of particular note here was the participants understanding that the decision to have minimalist slides and late posting was a positive decision by lecturers based in part on pedagogical principles. This frustrated the students at a number of levels. First was the sense that their learning styles may be different to standard research as this participant describes:

“it was kind of like that kind of paternalism of “this is how you should learn”, which I think they can be really frustrating because it’s not actually, it might be the best way to learn in terms of research or whatever but we learn differently” M1 FG1

But it was not just a potential mismatch between their learning style with research, but also on perceived intention. The students in this research interpreted the lack of detail and delay as obstruction by lecturers, reinforced by a lack of change following requests. The consequence was they perceived a lack of flexibility for them to use their own style and strategies to maximise their learning and indeed take ownership of their learning.

Timely posting of material generally was raised in relation to Virtual Learning Environments (VLE’s), the online portals where most learning resources are stored. The students suggested that a key strategy for most of them was to organise themselves early, so they knew what they needed to attend to well in advance. This was rendered impossible when materials were not posted in good time. But they also commented on their frustration when the location of materials changed, different modules posted things in different, seemingly random places or indeed the VLE itself changed.

Other areas participants highlighted included class numbers and room layout, all perceived to impact on their ability to engage. These students were aware that ‘putting things together’ is often an area that they have to work hard to achieve. Rather than facilitating that process, the academic practices created further obstacles which they were forced to navigate. As this participant suggests:

“So I think here they just limit it, you’ve got dyslexia, great, we’ll help you if you need to. Here’s extra time in exams, but that’s it. They (staff) don’t think about things like, so the presentations and what they look like and how visually it can affect. They don’t think about room sizes or, you know, one to one sessions or smaller group sessions and I think that is, is a big thing”. F3 FG2

Other points raised were the perception that timetables and group changes were haphazard which limited capacity to plan ahead or work with the people who understood their learning styles. The participants suggested that changes came with a lack of adequate explanation as to why they had occurred or an appreciation that such changes were disproportionately disruptive to some students with disabilities. For the participants in this study, this rather piecemeal approach to their disability was frustrating. They sensed that support was inconsistently applied which was in direct contrast to their 24-h experience of living with a disability.

For many this was a result of perceived staff ignorance of their diagnosis. While examples were given on placement, more often participants discussed clinical educators who themselves had specific learning difficulties. They were mostly seen as allies. Consequently, the call for more education was firmly directed towards University based staff.

“Personally in the physio department there’s not a big awareness of specific learning difficulties I don’t think…. My educator, I’d be on placement for two weeks, said I think you maybe should consider going and get tested, I’d been at university for two or three years now and I know, I get on with the lecturers and I give them pieces of work to have a look at, none of them advised that I get a test or anything like that “F6 FG3

This sense of staff ignorance was not universal, but like the adjustments was seen as piecemeal. It was chance if you had a tutor who recognised signs, made the effort to signpost appropriately, and who made suggestions on how placement could be approached. And chance was considered a problem.

Cumulatively, this lack of awareness, lack of consideration of the on-going challenges of studying with a disability and adjustments that were only periodically considered had one overriding impact on the participants; an impedance to them managing their own situation. This disempowerment lay at odds with a clear sentiment across the participants in this study, that they were aware of their own dis/abilities and they were very willing to take ownership of their personal situation.

## Discussion

The narratives from this group of students with disabilities highlight a number of aspects which they associate with potentially impeding their success.

Early identification and orientation to the physiotherapy context facilitated recognition, understanding and development of strategies in order for these students to succeed as the independent professionals they aspired to be. When successful, these processes had the potential to shift the participants’ identity resulting in confirmation of their skills and capabilities. Clouder et al. [15] note that students with a disability often have strong agency and a desire to engage. This was also evident in this study as the participants demonstrated how an understanding of self, resulted in a desire to reject the deficit view of their disability and affirm their positive skills and attributes, possibly reflecting an emerging affirmation model [24]. The findings suggest that the process of transformation and the students’ confidence in engagement was hampered as a consequence of inadequate and inconsistently applied systems and staff ignorance. Therefore informed, proactive, timely and responsive systems that are equitably delivered to maximise the students’ potential for self-discovery and development are important and may impact positively on their successful progression in the course.

The further findings from this study illustrate some of the challenges of the learning environment and practices for students. Interestingly, issues identified were most frequently situated within the university arena rather than the clinical environment which is surprising, given that previous literature in the field has largely focused on the practice environment [8, 12, 13, 18, 19, 23].

One issue relevant to the clinical and university settings was ambiguity in disclosure. Disclosure of their disability was rarely discussed by the participants in relation to potential prejudice as described by other authors [8, 30–35]. Rather, in-line with Opie and Taylor [13] concern was situated once more in ignorance. On one side the students were unclear how their disability may impact on placement and as a consequence questioned the need for disclosure. On the other, the students perceived their educators both at university and on placement to be ignorant of specific disabilities and therefore disclosure may not be met with a supportive response. This insight suggests that the ambiguity around disclosure requires more nuanced examination and perhaps further exploration of qualified staff awareness of disability (particularly specific learning disabilities) is needed.

In relation to the university environment, numerous examples were highlighted by the participants indicating how their engagement and assessment potential were hampered. These raise some significant questions about what might be done to make the education experience more inclusive and what adjustments might be considered reasonable alongside the expectations of professional behaviour and competence. Understanding these opportunities may assist in facilitating engagement and subsequent assessment success.

One example noted by the students was the inaccessibility of the learning environments, whether through lecture format and notes, class sizes and structure or the virtual learning sites. A number of possible contributing factors were implied for this apparent lack of consideration of the wider features of education delivery such as: i) lack of familiarity of staff with principles of inclusive education (as described by Hockings [21]), ii) a lack of willingness to deliver on it or alternatively iii) a mismatch between the students’ expectations of specific delivery methods (e.g. introductory lectures) and the pedagogical considerations by the faculty. What was explicit in this study is that standardised and inflexible teaching and learning practices actively impede the students’ engagement, autonomy and management of self, a view supported by related literature and linked to success [20, 26, 36, 37]. Furthermore, there appeared to be little opportunity for effective dialogue with academic staff to redress this.

A potentially more complex example was that of a lack of consideration and flexibility in practical examinations. The ability of these assessments to reflect clinical reality was questioned by the students who found the clinical environment to be more accommodating of alternative ways of processing and delivering information. This contradiction in experience raises some key questions about the purpose and process of practical assessments.

While personal fitness to practice was not expressly raised in this study, the implied necessity of clinical competence suggests the concept of ensuring students are fit to practice may influence faculty staff decision making about flexibility within practical assessment. For instance, extra time adjustments may be given for reading preparation but not within situations that apparently replicate a clinical encounter, which is similar to guidance for medical education [38]. However, this neither considers the iterative real time clinical reasoning processes that occur during practical examinations that were often described by the participants in this study. Nor does it potentially reflect many therapeutic clinical encounters in which the assumption of immediate and time restricted response (for purposes of safety) are not necessarily required. Discrepancy is also noted in students’ descriptions of proactive use of templates to support their organisation and decision-making while on placement which are explicitly not permitted within university assessments. Interestingly, alternative formats for students to respond to clinical questions, such as use of paper and diagrams, is recommended by the General Medical Council [38].

As a consequence, the very purpose of reasonable adjustments is questioned and a dilemma of prioritising safety over effectiveness is created (also reported in nursing education [39]). A focus on examination competence that does not reflect clinical need and competence suggests an approach to university assessment based on homonormativity and ableist processes. This limits students’ potential and autonomy, decreases their capacity to explore and be appropriately assessed on strategies they could use in practice, but also inappropriately homogenises all clinical situations. Eaterbrook et al. [20] and Bulk et al. [36] note that there needs to be greater clarity on necessary competencies and appropriate inflexibility alongside acceptance of where approaches and structures can be changed. A more considered approach to practical based assessments which involves closer dialogue with both clinical partners and students would result in an improved capacity to align assessment with clinical reality and assist visibility of decision making. This is particularly required given that currently the students perceive that the support they receive on placement is more appropriate and flexible and utilises their enhanced skill set rather than remaining in a deficit model.

A key aspect of these findings is that understanding these students’ experiences and the implication of education has broad relevance. Their narratives are a challenge not only to how education providers engage with students with disabilities, but how they engage with educating all students. Shakespeare [40] calls for a deconstruction of an assumed normality and in highlighting discontinuities in their experience, which they note impacts on all students, the students in this study echo that call.

The overall sense of these data is that these students are caught within a focus and indeed the language of deficit by a system that they perceive prioritises normalisation, while they indicate a desire to explore, affirm and express their able diversity. Adjustments for them were not seen as a concession to normalise but an opportunity to learn and perform to the best of their ability and reflected their experience in the clinical environment. This lay in contrast to what they perceive as the fractured institutional processes which appeared to remain in concessionary structure, but specifically one that only pertained to particular tasks, reading and written assessments. As a consequence, the inclusivity of the curricula and its delivery may be questioned, and the opportunities to celebrate able diversity need to be reconsidered.

This study has number of strengths and limitations which are important to consider. The limited representation of different disabilities, specifically physical and visual is acknowledged. Likewise, the specific locality of the study and its focus on two institutions may limit that relevance of the findings to a wider audience, however readers are encouraged to consider similarities to their own context. To counter some of these limitations a researcher with a disability was included during all stages, care was taken to encourage open conversations within the focus groups and the rigour of the analysis and the member checking of initial summaries gives strength to the depth and direction of the analysis.

## Conclusion

These focus groups with physiotherapy students with a range of different specific learning difficulties and other long term mental health disorders, has demonstrated that studying physiotherapy involves complex dynamics, which the students have to learn to navigate. The students navigate factors that include ignorance of their conditions within the profession and educational institutions which results in often haphazard routes to diagnosis, ambiguity with disclosure, and critically, both a lack of continuity in the institutions approach to support and inadequate consideration of the impact of the learning environment, assessment and resources. While the students accepted that these concerns often relate to all students, they felt that they disproportionately impacted on those with disabilities.

As a result, the students felt somewhat disempowered by structures and processes throughout their course. These hurdles also impede use of their self-management strategies that they have worked hard to develop and wish to implement.

This was a small study based in a particular region of the UK. However, given the dearth of research to date within physiotherapy, it is an important step forward. Specifically, this study extends the literature in this area by highlighting the issues experienced within the university setting which have been under-reported in physiotherapy. This particularly relates to the concepts of and limitations to reasonable adjustments. It further highlights the ambiguity of disclosure, indicating a need for further exploration.

There is a need for more studies looking at the experience and attainment of students with disabilities nationally, with a focus on the factors that impact on their learning experience and successful strategies to facilitate that process. Specific focus on the transition from University to clinical environments is also warranted.

## Implications

Students have a wealth of knowledge based on their experiences of living with a disability while undertaking pre-registration physiotherapy studies. The insights they raise pose a number of challenges to education providers both in academic and clinical environments. While caution is appropriately raised in regard to generalisations for what is a very heterogeneous group, some general considerations can be raised from this study. These include; consistent and accessible signposting to facilitate early self-understanding and recognition of strategies and skills; increase staff awareness of individual experiences of disability and how this can impact on learning in different environments; critical examination of the justifications for what is considered ‘reasonable’ in terms of adjustments; improved interaction between clinical and university based education providers to share understanding and expectations in relation to adjustments and clinical competence; and a reconsideration of inclusive education that creates opportunity to allow and celebrate able diversity.

## Supplementary information

**Additional file 1.** Topic Guide.

## Data Availability

The datasets used and/or analysed during the current study are available from the corresponding author on reasonable request.

## Abbreviations

ADD: Attention deficit disorder
ADHD: Attention deficit hyperactive disorder
HEI: Higher education institutions
NHS: National Health Service
OSCE: Objective structured clinical examination
UK: United Kingdom
VLE: Virtual learning environment
