# Supplementary material for: Students with specific learning disabilities experiences of pre-registration physiotherapy education: a qualitative study
Source: BMC Med Educ. 2019 Dec 31;20:2. doi: 10.1186/s12909-019-1913-3 (PMC6938602; doi:10.1186/s12909-019-1913-3)
Supplement: Supplementary file 1 — Additional file 1. Topic Guide. [file 12909_2019_1913_MOESM1_ESM.docx]

**Topic guide**

**Introduction**

Recap

- Purpose of focus group – recap objectives
- Ground rules – equal voice, not prejudicial, confidentiality
  length – around 90 minutes
- Right to withdraw
- Role of facilitator and assistant
- Mobile phones on silence
- Audio recording device

**Introductory questions**

- Participants will be provided with demographic sheet based on HESA categories for disability and asked to record/share with the group
  - How does the categorization match with their self –perception/description
- Did your disability play any part in your decision or process in choosing physiotherapy as a career? If so how?

**Activity 1**

- Post-it note activity in relation to experience of studying Physiotherapy with a disability (from first day until now)
  - Green - positive things
  - Red - things that would enhance
  - White – anything that is specific to disability (positive and not so great)

Students have 10 minutes to write and then share and **discuss**. Can ask them to group post-its to identify common areas and points of differences.

Prompts

- check meaning, relevance of WP influence
- How they overcame challenges
- Check for enhancements due to WP influence

**Activity 2**

- Review relevant data from previous regional study. Ask what the participants think of the findings? How does it make them feel? (open questions) What do you think may explain the findings?

Prompts

- - Check personal, environmental, institutional factors
- Were you expecting these results? Why?
- If not what were you expecting and why?

**Activity 3**

- What do you think could be done to redress this attainment gap? Put responses on flip charts around the room to facilitate sharing
  - Who?
  - Do what?
  - Where?
  - How?
  - When?
  - Agree prioritization – what must be done, what should be done, what could be done.

**Conclusion**

Finish with summary of main findings and recap on importance of the FG and their contribution

Remind them of potential to hear results.

End
